# Cerebro-Spinal Meningitis

**Published:** 1900-03-17

**Authors:** G. Hutchinson Milnes

**Affiliations:** Physician Derbyshire Royal Infirmary; Consulting Physician Derbyshire Hospital for Women.


### Hospital Clinics and Medical Progress.

### CEREBRO-SPINAL MENINGITIS.

By G. Hutchinson Milnes, M.D.Camb., Physician Derbyshire Royal Infirmary; Consulting Physician Derbyshire Hospital for Women.

(*Continued from page* 376.)

Whenever a person is suddenly taken profoundly ill with some affection of the central nervous system, and has at the same time a high temperature and a slow pulse (say under 90), think of cerebro-spinal meningitis. The skin usually presents a dusky aspect, the eyes are heavy and suffused, conjunctiva inclined to be pink. The pupils are usually dilated, but act readily. The patient is peevish, obeys orders slowly, and resents interference. Eruptions on the skin are frequently present. Herpes labialis is rather more common in this disease than in pneumonia. Purpura hæmorrhagica and petechial spots are so frequent that it has been called the spotted fever. It is then very hard to distinguish from typhus. There is almost always headache, usually occipital, and soon the typical symptoms of the disease develop, to wit, pain and tenderness and stiffness at the back of the neck. Retraction of the head, sometimes extreme. Pain, stiffness, and tenderness of the muscles of the back, which may be so extreme as to cause opisthotonos, but usually stops short at orthotonos. These symptoms referring to the neck and back are seldom entirely absent, but may be slight and transitory. The stiffness may spread to the limbs. Hyperæsthesia of the trunk is usually present in the early stage. So pronounced was abdominal hyperæsthesia in my first case that appendicitis and peritonitis was strongly suspected. Anæsthesia is much less common unless profound coma has been established. It was, however, markedly present in a case I saw a few weeks ago, by the kindness of Dr. Benthall, where the patient, who was nothing more than lethargic, had allowed a hot-water bottle to raise a large blister on the thigh without perceiving it. Paralyses are rare, except of the ocular muscles and of the face. Delirium, usually active, is seldom entirely absent from any case; it usually alternates with periods of clear-headedness and lethargy. The principal feature of my second case in the early stages was maniacal delirium, alternating with profound stupor.

As the case progresses the tendency to coma becomes more marked, until for several days before death this is continuous and increasingly profound. Convulsions do not as a rule occur, but spasmodic squint is sometimes seen. Cheyne-Stokes' respiration, and irregularities of pulse and respiration are fairly frequent. The blood presents a constant, moderate, but well-marked leucocytosis, and this is important, as it serves as a means of diagnosis from typhoid fever, which this disease often closely resembles.

The prognosis is variable, the death-rate ranging in various epidemics from 20 to 75 per cent.

The treatment has been equally varied and futile; it is best to carry it out in accordance with general methods. My first case well illustrates the failure of, at any rate, internal antipyretics. Operative treatment will be considered later on.

Three varieties of this disease are described: (1) The fulminating, where death with coma or other profound nervous affection occurs within 24 or 48 hours. Sometimes the duration is as short as five hours, and even in that short space of time the cardinal symptoms of the disease have been noted and a correct diagnosis made. (2) The ordinary class of case presenting such symptoms as I have described, and running a course of from two to three weeks. Other cases occur in which the symptoms are mild throughout, and convalescence is established by the end of the third week. These must not be confounded with the third type of the disease. (3) The abortive, in which although the onset is violent and everything presages a very severe attack, the symptoms suddenly disappear after about four days, and a rapid convalescence ensues.

### Case I.

Nelly Kerry, aged 21. Admitted May 19th, 1897, had an illness stated to be pleurisy six months ago. Has recently suffered mental distress through love affairs. On the evening of the 17th became suddenly ill, pain in left iliac region, headache, fever, and vomiting; symptoms have lasted ever since. On admission face flushed, restless and tossing about, not delirious, temp. 101·2, pulse 92, full and strong, tongue thick dirty fur, resents examination, pain in abdomen and back of head, tendency to retraction of head and tenderness of the back of head and neck, all organs appear normal, hyperæsthesia of trunk, especially abdomen, pupils normal and act to light.

20th.—A rigor. Pulse 86, rigidity of legs and back transient. Symptoms continued until the 22nd, when she became quieter. Temperature varies from 103 deg. to 105 deg.

24th.—Quiet and rational, no pain in head, abdomen still hyperæsthetic.

26th.—Active delirium, no rigidity of trunk or limbs.

27th.—Delirium gave way to deep sleep. Resents examination.

28th.—Stupor. Resents examination.

29th.—Stupor. Increased hyperæsthesia, abdomen retracted and rigid, Tâche cerebrale present, ophthalmoscope reveals nothing abnormal. After this date, gradually passed into a state of profound coma, with a pulse rate up to 120; pupils became sluggish, but their reaction was never abolished.

On May 30th an attempt to reduce the temperature by ice pads failed.

On June 1st doses of antipyrin and salicylate of soda reduced the temperature in 12 hours from 101·4 to 97 deg. No other symptoms were relieved. Cheyne-Stokes' breathing set in. She died at three p.m.

Post-mortem 22 hours after death. No organs presented any interesting change, except the brain and spinal cord.

**Brain.**—Vessels of the pia mater all over the vertex gorged with fluid blood. The whole base of the brain as far forward as the optic commissure was covered with yellow lymph confined in the sub-arachnoid space, which contained also a quantity of turbid fluid. The brain substance was very firm, the cortical grey matter a deep colour, and puncta vasculosa well marked.

The third and lateral ventricles were enlarged, and contained much turbid fluid. The ventricular walls were softened, the body of the fornix was unrecognisable, and the choroid plexus was matted together by a profuse deposit of yellow lymph. This condition was also found in the fourth ventricle.

**The Spinal Cord.**—Dura mater distended with turbid fluid were contained in the arachnoid space. In the cervical region some ecchymosis in the dura mater. In the dorsal and lumbar regions much lymph in the pia mater around the cord, the substance of which appears firm and healthy and not congested. Varied methods of treatment were tried in this case, but none appeared to have any effect.

Case II.

Henry Johnson, aged 18. Admitted Sept. 19th, 1899, with a certificate saying he had influenza with delirium. Illness began two days ago; the onset was very sudden. On admission he was extremely violent and delirious, but a dose of bromide and chloral quieted him. Next day, September 20th, pulse 64; face flushed; herpes on lips. Examination detected nothing abnormal in any organ. Towards night he became very violently delirious, and the same draught again quieted him. He was ordered daily a large dose of calomel and a turpentine enema. His condition alternated between violent delirium and profound stupor until September 24th, during which time the pulse was slow and irregular. There was great rigidity of the back of the neck and of all the muscles of the back, with tenderness, producing marked opisthotonos. From this date the symptoms rapidly subsided, and on October 13th he was well.

*Remarks.*—These two cases fairly illustrate the symptoms described in the former portion of this paper, as being typical of cerebro-spinal meningitis. The symptoms in the second case were more violent than those of the first; the possibility of recovery appeared more remote. The treatment was more actively carried out. Indeed, by a misconception of her orders on the part of the sister, the treatment was even more heroic than was intended by the physician. For the latter having ordered a turpentine injection to be given daily, it was administered every four hours for the space of four days before the mistake was detected. During this period the relief of grave symptoms was well marked. Some years ago I had under my care a young man suffering from cerebral hæmorrhage. During the progress of the case, when convalescence seemed to be established, a gradual rise of temperature took place, until in the course of four or five days it had reached 104 deg. It was thought to be due to inflammatory changes around the still unhealed lesion in the brain. A daily enema of turpentine was ordered, and the temperature at once began to subside in the same deliberate manner in which it had risen. The turpentine injections were stopped, and the temperature remained normal for some days, when it again rose in the same manner as before. As I was puzzled to account for this state of affairs, the sister pointed out that the previous subsidence of temperature coincided with the administration of the turpentine. This was therefore resumed; the temperature again subsided, the turpentine was continued for a considerable period, and complete recovery eventually took place. I am not concerned to maintain that turpentine may be expected to exert a specific influence over inflammatory diseases of the central nervous system, but in affections, to contend with which all known remedies have hitherto proved so futile, it seems reasonable to note its apparent effect in these two cases, and to give it a further trial.

